# Intrinsically disordered regions are abundant in simplexvirus proteomes and display signatures of positive selection

**DOI:** 10.1093/ve/veaa028

**Published:** 2020-05-10

**Authors:** Alessandra Mozzi, Diego Forni, Rachele Cagliani, Mario Clerici, Uberto Pozzoli, Manuela Sironi

**Affiliations:** v1 Scientific Institute, IRCCS E. MEDEA, Bioinformatics, Bosisio Parini 23842, Italy; v2 Department of Physiopathology and Transplantation, University of Milan, Milan 20090, Italy; v3 Don C. Gnocchi Foundation ONLUS, IRCCS, Milan 20148, Italy

**Keywords:** HSV-2, simplexviruses, positive selection, intrinsically disordered regions (IDRs), virus–host interactors

## Abstract

Whereas the majority of herpesviruses co-speciated with their mammalian hosts, human herpes simplex virus 2 (HSV-2, genus *Simplexvirus*) most likely originated from the cross-species transmission of chimpanzee herpesvirus 1 to an ancestor of modern humans*.* We exploited the peculiar evolutionary history of HSV-2 to investigate the selective events that drove herpesvirus adaptation to a new host. We show that HSV-2 intrinsically disordered regions (IDRs)—that is, protein domains that do not adopt compact three-dimensional structures—are strongly enriched in positive selection signals. Analysis of viral proteomes indicated that a significantly higher portion of simplexvirus proteins is disordered compared with the proteins of other human herpesviruses. IDR abundance in simplexvirus proteomes was not a consequence of the base composition of their genomes (high G + C content). Conversely, protein function determines the IDR fraction, which is significantly higher in viral proteins that interact with human factors. We also found that the average extent of disorder in herpesvirus proteins tends to parallel that of their human interactors. These data suggest that viruses that interact with fast-evolving, disordered human proteins, in turn, evolve disordered viral interactors poised for innovation. We propose that the high IDR fraction present in simplexvirus proteomes contributes to their wider host range compared with other herpesviruses.

## 1. Introduction

Herpesviruses (order *Herpesvirales*) constitute a diverse family of enveloped, double-stranded DNA viruses that infect a wide range of animals, usually resulting in life-long infections (McGeoch, Rixon, and Davison 2006). Herpesviruses have complex genomes, which characteristically contain regions of unique sequence flanked by direct or inverted repeats. Although herpesviruses express non-coding RNAs and miRNAs, protein-coding regions occupy the great majority of their genomes (McGeoch, Rixon, and Davison 2006).

Nine herpesviruses, all of them belonging to the *Herpesviridae* family, naturally infect humans: herpes simplex viruses 1 and 2 (HSV-1 and -2), varicella-zoster virus (VZV), Epstein–Barr virus (EBV), human cytomegalovirus (HCMV), human herpesviruses 6A and 6B (HHV-6A and -6B), human herpesvirus 7 (HHV-7), and human herpesvirus 8 (HHV-8, also known as Kaposi’s sarcoma-associated herpesvirus). These viruses are among the most successful human pathogens in terms of global distribution, persistence in the host, and transmissibility. Most adults are infected with at least one herpesvirus and the seroprevalence of some of these viruses is as high as 90 per cent in human populations (Balfour et al. 2013). Primary infection of immunocompetent human subjects generally results in a mild disease, although severe consequences may develop in specific groups of individuals. For instance, intra-uterine HCMV infection is the leading infectious cause of deafness and intellectual disability in children (Manicklal et al. 2013), whereas HSV-1, -2, and VZV are common etiologic agents of non-epidemic acute encephalitis in Western countries (Venkatesan 2013). When contracted at birth, human simplexviruses (HSV-1 and -2) can also cause neonatal invasive infection (Whitley 2004).

Viruses related to HHVs have been described in non-human primates (NHPs; Tischer and Osterrieder 2010). In these animals, seroprevalence can be very high and, similarly to what is observed in humans; infection can either cause a mild disease or can be asymptomatic (Tischer and Osterrieder 2010). In analogy to observations in other mammalian and non-mammalian hosts, herpesvirus infection is remarkably species-specific in primates, with most viruses naturally infecting a single host. Nonetheless, cases of cross-species transmission have been described (Eberle and Jones-Engel 2017). One of these refers to the macacine herpesvirus 1 (also known as B-virus), a simplexvirus indigenous in Asiatic macaques. In its natural host, B-virus infection is almost asymptomatic, but transmission to humans results in a severe, often fatal form of encephalomyelitis (Tischer and Osterrieder 2010; Eberle and Jones-Engel 2017). Likewise, infection of New World monkeys with HSV-1 is almost invariably fatal (Tischer and Osterrieder 2010).

Not only these examples highlight the potential zoonotic threat posed by herpesviruses but they also point to long-standing host–virus association. Indeed, the phylogenetic relationships among hepeviruses tend to mirror those among their hosts, indicating that viral lineages frequently arose through co-speciation with host lineages (McGeoch, Rixon, and Davison 2006). Among HHVs, though, an exception to this general tendency is represented by HSV-2. In fact, this virus most likely originated around 1.6 million years ago from the cross-species transmission of Chimpanzee herpes virus 1 (PanHV-3) to an ancestor of modern humans (Severini et al. 2013; Wertheim et al. 2014; Underdown, Kumar, and Houldcroft 2017).

Host-shift events are often accompanied by bursts of positive selection, as the virus adapts to infect and to be efficiently transmitted in a new species (Longdon et al. 2014; Sironi et al. 2015). We thus exploited the availability of the PanHV-3 genome, as well as of several HSV-2 sequences, to search for adaptive variants that arose or increased in frequency after the cross-species transmission event. Our results indicate that positive selection mainly occurred in HSV-2 protein regions that are intrinsically disordered (intrinsically disordered regions, IDRs)—that is, domains that do not adopt compact three-dimensional structures (Dyson and Wright 2005). We also show that, compared with other herpesviruses, IDRs are particularly abundant in the proteomes of primate-infecting simplexviruses and that the average extent of disorder in viral proteins tends to parallel that of the host interactors.

## 2. Methods

### 2.1 Sequences and alignments

HSV-2 genome sequences were retrieved from the NCBI (http://www.ncbi.nlm.nih.gov/, last accessed 20 January 2020) database. A list of accession IDs is reported in Supplementary Table S1.

Genome sequences were chosen to be representative of the genetic diversity of circulating HSV-2 strains. The pool of HSV-2 genomes included an equal number of strains sampled in different geographic areas. Only isolates that were directly sequenced with no (or very limited) *in vitro* passages were included. The genome sequence of the Chimpanzee alpha-1 herpesvirus (PanHV-3, NC_023677) was also retrieved from NCBI.

For each viral genome, we retrieved coding sequences of all annotated open reading frames (ORFs). For non-annotated genomes, ORFs were deduced by whole genomes alignments performed with Progressive MAUVE 2.3.1 (Darling et al. 2004; Darling, Mau, and Perna 2010), and orthology was inferred according to MAUVE attribution.

Fully or partially overlapping ORFs were merged (i.e. *UL26/UL26A*), completely removed (i.e. *UL15/UL16/UL17)*, or partially analyzed (i.e. *UL13/UL14, US8/US8A*, and *US10/US11*), depending on whether they are translated in the same frame or not. Likewise, the *UL29*, *UL30*, and *UL39* genes were excluded from the analysis because most HSV-2 strains carry fragments deriving from recombination with HSV-1 (Burrel et al. 2017; Casto et al. 2020).

For each gene, alignments were generated using MAFFT (Katoh and Standley 2013), setting sequence type as codons; unreliably aligned codons were filtered using GUIDANCE2 (Sela et al. 2015) with a codon score of 0.90 (Privman, Penn, and Pupko 2012). The resulting alignments were manually inspected.

### 2.2 Analysis of selective patterns in HSV-2

Analyses were performed with gammaMap that uses intra-specific variation and inter-specific diversity to estimate, along coding regions, the distribution of selection coefficients (γ). In this framework, *γ* is defined as 2PN_e_s, where *P* is the ploidy, *N*_e_ is effective population size, and *s* is the fitness advantage of any amino acid-replacing derived allele (Wilson et al. 2011).

For each gene, the corresponding coding sequence of PanHV-3 was used as the outgroup.

We assumed θ (neutral mutation rate per site), *k* (transitions/transversions ratio), and *T* (branch length) to vary within genes following log-normal distributions, whereas *P* (probability of adjacent codons to share the same selection coefficient) following a log-uniform distribution. For each gene, we set the neutral frequencies of non-STOP codons (1/61). For selection coefficients, we considered a uniform Dirichlet distribution with the same prior weight for each selection class. For each gene, we performed two runs with 100,000 iterations each and with a thinning interval of ten iterations. Runs were merged after checking for convergence and sites showing a cumulative probability higher than 0.75 of having γ ≥ 1 (Quach et al. 2013) were defined as positively selected.

HSV-2 genes were classified as ‘core’ or ‘non-core’ following the characterization by Davison (2007).

The dN–dS parameter was calculated using the single-likelihood ancestor counting method (Kosakovsky Pond and Frost 2005).

### 2.3 Analysis of intrinsic disorder in herpesvirus and human proteins

IUPred2 (https://iupred2a.elte.hu/plot, last accessed 20 January 2020; Meszaros, Simon, and Dosztányi 2009; Mészáros, Erdos, and Dosztányi 2018; Dosztanyi 2018) was used to predict the fraction of disordered residues (score > 0.5) in the proteomes of HSV-2, of viruses in the *Simplexvirus* genus, as well as of other HHVs spanning all the three *Herpesviridae* subfamilies (*Alphaherpesvirinae*, *Betaherpesvirinae*, and *Gammaherpesvirinae*, Supplementary Table S2). The whole proteome of each viral species was retrieved from NCBI database.

A long disorder prediction type was selected for all analyses. We defined as IDR, a protein region with at least thirty consecutive amino acids showing a IUPred2 score > 0.5 (Ward et al. 2004; Dosztanyi 2018).

The binomial test was performed by counting, for the sixty-seven analyzed ORFs, the number of disordered regions carrying at least one positively selected site (to avoid the non-independence among nearby sites), the total number of all regions (disordered or not) carrying a positively selected site, and the fraction of IDR length compared with the total length of the proteome.

Human–virus protein–protein interactions were obtained from the virus Mentha database (https://virusmentha.uniroma2.it/, last accessed 20 January 2020; Calderone, Castagnoli, and Cesareni 2013; Calderone, Licata, and Cesareni 2015). As few interactions were available for HSV-2, and because HSV-1 and -2 are closely related, we merged interactors of both viruses. Interactors of HHV-6B and VZV were not analyzed, as few data were available. IDRs for human proteins were calculated as for viral molecules.

HSV-1, -2, and VZV ortholog core genes were retrieved from Davison (2007).

All statistical tests were performed in the R environment (version 3.6.2, http://www.r-project.org, last accessed 7 January 2020).

## 3. Results

### 3.1 IDRs in HSV-2 proteins display abundant signals of positive selection

As mentioned earlier, HSV-2 is phylogenetically related to PanHV-3, as it is thought to have originated from the cross-species transmission of this latter virus to an intermediate host, possibly *Paranthropus boisei*, eventually reaching an ancestor of modern humans (Underdown, Kumar, and Houldcroft 2017). To investigate the selective events that accompanied the adaptation of HSV-2 to our species, we applied a population genetics–phylogenetics approach. Specifically, we used the gammaMap program (Wilson et al. 2011), that leverages intra-species variation and inter-species diversity to estimate the distribution of fitness effects (i.e. selection coefficients, γ, expressed as discrete categories from −500 to 100) along coding regions. In practical terms, *γ*  values can be considered a measure of the fitness consequences of new non-synonymous mutations. Thus, we used the PanHV-3 sequence as an outgroup and we analyzed a representative phylogeny of fifty-three HSV-2 strains derived from clinical isolates sampled worldwide (Supplementary Table S1); among these, we included strains belonging to the recently identified African HSV-2 lineage (Burrel et al. 2017). For each HSV-2-coding gene annotated in the reference HG52 strain, orthologs were retrieved and aligned (see Section 2).

**Figure 1.**
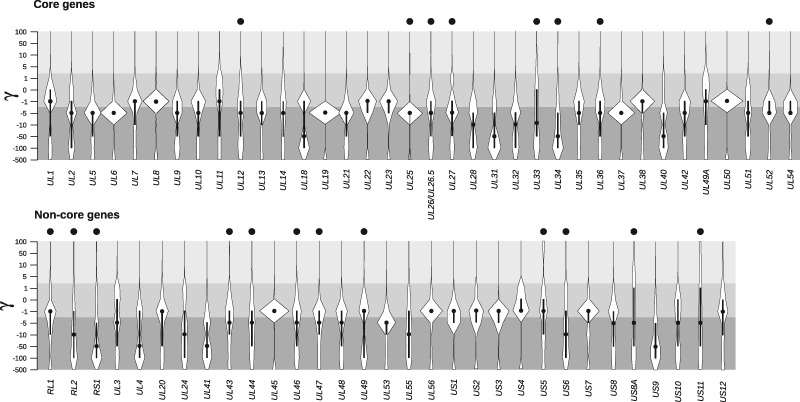
Population genetics–phylogenetics analysis of HSV-2 genes. Violin plots (median, black dot; interquartile range, black bar) of selection coefficients for HSV-2 core and non-core genes. The gray shading denotes different degrees of constraint based on selection coefficients (γ  ≤  5, moderately or strongly deleterious, inviable; −1 ≤ γ  ≤  1, neutral or weakly deleterious/beneficial; γ ≥ 5, moderately or strongly beneficial). Black dots above the plots indicated genes in which positively selected sites were detected by gammaMap (see Section 2).

After discarding overlapping ORFs and genes (*UL29*, *UL30*, and *UL39)* deriving from recombination with HSV-1 (Burrel et al. 2017; Casto et al. 2020), the distribution of selection coefficients was estimated along sixty-seven ORFs (Fig. 1). These included both ‘core’ genes that are shared by all herpesviruses, and ‘non-core’ genes, which are specific for the *Simplexvirus* genus or for HSV-1/-2 only.

In general, most median values of γ were comprised between −10 and −1, both for core and for non-core genes, indicating that the majority of sites are subject to weak purifying selection. Only eight genes evolved under a stronger negative constraint (median γ = −50). Some of these latter code for proteins playing a key role in viral replication and spread (*UL31*, *UL34*, *UL40*, *UL41*, *UL4*, and *US9*) or for fundamental viral structural components (*UL18*).

To identify signals of positive selection, we estimated codon-wise posterior probabilities for each selection coefficient. Specifically, we defined a codon as positively selected if its cumulative posterior probability of γ ≥ 1 was > 0.75. According to these criteria, we identified positively selected sites in twenty genes, eight cores (fraction selected= 22.2%), and twelve non-core (fraction selected = 38.7%; Fig. 1 andSupplementary Table S3).

**Figure 2.**
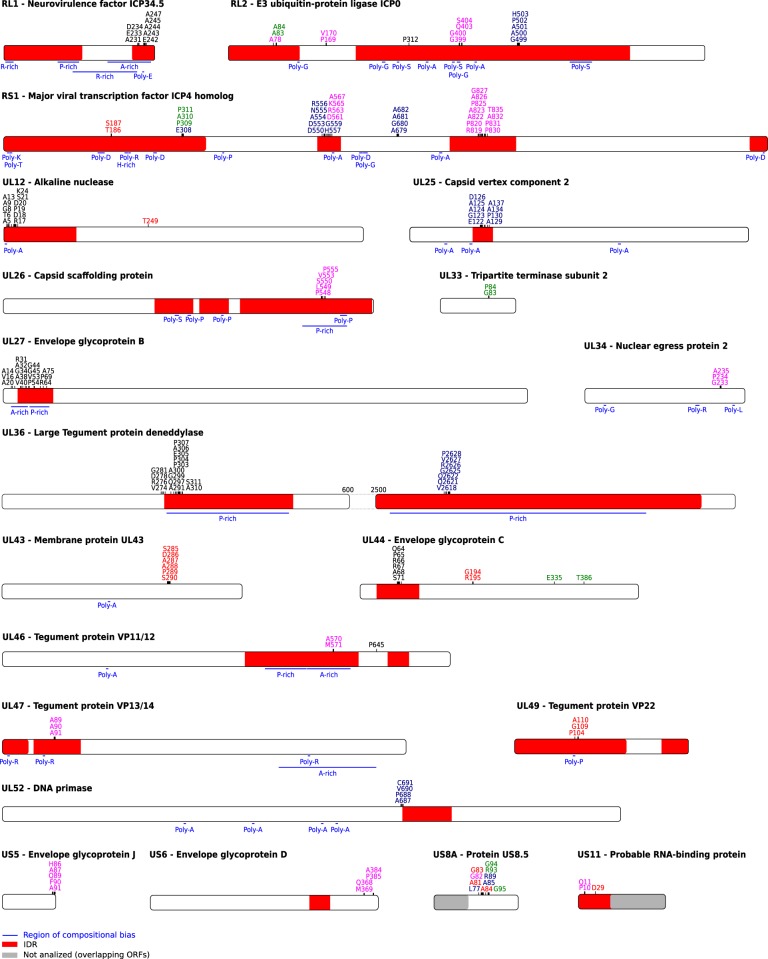
Distribution of positively selected sites. Sites that were detected by gammaMap as positively selected were mapped onto HSV-2 proteins together with the location of IDRs (red). Sites are color coded based on their highest posterior probability of γ (1, black; 5, blue; 10, green; 50, red; 100, magenta). Overlapping coding regions not considered in gammaMap analysis are in gray. The compositional biased region is also indicated along the protein sequence (blue lines). Positions refer to the reference HG52 strain (NC_001798).

**Figure 3.**
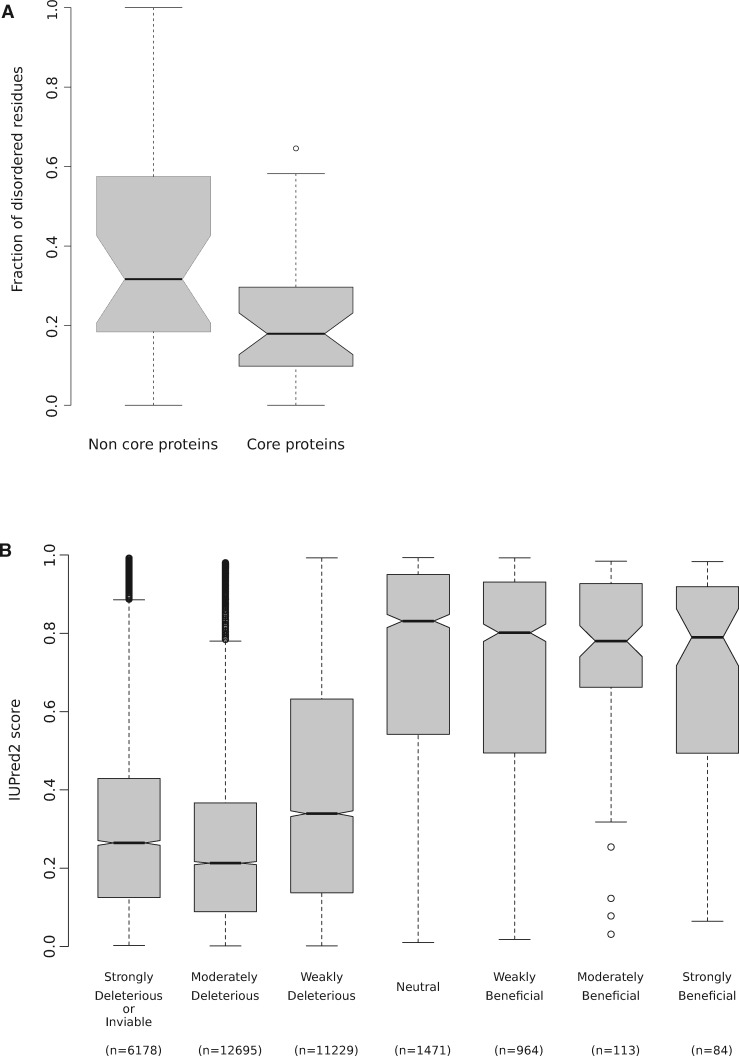
Intrinsically disordered residues in HSV-2 proteins. (A) The fraction of disordered residues of each HSV-2 non-core or core protein is plotted as boxplot. (B) IUPred2 score calculated for each residue of all HSV-2 protein is plotted as boxplot. Residues are grouped based on their highest posterior probability of γ, as calculated by gammaMap. Selection coefficients were defined as: strongly deleterious/inviable (γ ≤ −50), moderately deleterious (γ = −10 or γ = −5), weakly deleterious (γ = −-1), neutral (γ = 0), weakly beneficial (γ = 1), moderately beneficial (γ = 5 or γ = 10), and strongly beneficial (γ = 50 or γ = 100).

Analysis of positively selected sites indicated that ∼29 per cent of them map to regions with biased amino acid composition (e.g. proline-rich, alaninine-rich; Fig. 2). Because such regions are often disordered, we investigated whether positive selection is more likely to occur in IDRs. Thus, the presence of IDRs was predicted with IUPred2 (Mészáros, Simon, and Dosztányi 2009; Mészáros, Erdos, and Dosztányi 2018; Dosztanyi 2018) for all HSV-2 proteins we analyzed with gammaMap. We found that the large majority of selected sites fall within IDRs. In particular, we defined IDRs as regions with at least thirty consecutive residues with a IUPred2 score higher than 0.5 (Ward et al. 2004; Dosztanyi 2018). Statistical analysis indicated that IDRs are significantly more likely to harbor at least one positively selected site than expected based on their relative proportion in HSV-2 proteins (Binomial test, *P* = 2.3 × 10^−07^, see Section 2). This finding is in line with the fact that a higher fraction of non-core genes are positively selected, as their encoded proteins harbor more disordered residues compared with proteins encoded by core genes (Wilcoxon rank sum test, *P* = 0.023; Fig. 3A).

Previous analysis of IDRs, mostly from cellular organisms, indicated that these regions frequently display signatures of positive selection and that they are subject to weaker selective constraint than ordered regions (Brown, Johnson, and Daughdrill 2010). We thus compared the disorder score of HSV-2 protein residues with different selection coefficients (ranging from strongly deleterious or lethal to strongly beneficial). Results indicated a clear-cut difference in the distribution of disorder scores, with most constrained sites being preferentially located in ordered protein regions (Fig. 3B).

gammaMap models the allele frequency spectrum (conditioned on the ancestral allele) within a population and combines it with a model of the substitution process between species. The method thus intrinsically differs from approaches based on the comparison of the non-synonymous (dN) and synonymous (dS) substitution rates, which are best-suited to study inter-species diversity (i.e. long-term evolutionary processes; Kryazhimskiy and Plotkin 2008). As a comparison, for the twenty genes with at least one positively selected site detected by gammaMap, we calculated dN–dS. This metric was preferred over the conventional dN/dS ratio because it is not rendered to infinite for dS values equal to 0. As expected, virtually no correlation was observed between dN–dS and γ values (Kendall’s rank correlation, tau = 0.048, *P* = 4.4 × 10^−10^; Supplementary Fig. S1).

### 3.2 Primate simplexvirus proteomes are particularly rich in IDRs

**Figure 4.**
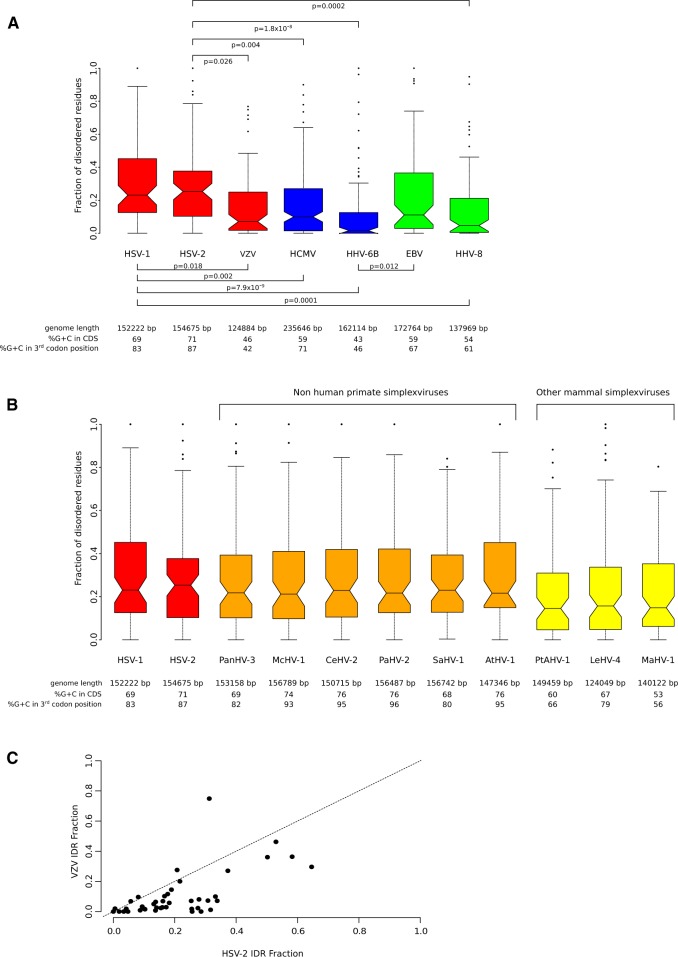
Intrinsically disordered residues in herpesviruses*.* (A) Boxplot representation of the fraction of disordered residues for HHV proteins. The genome length of each virus is also reported, along with the percentage of G + C content calculated for the whole-coding sequence or considering the third codon position only. Viruses are colored based on their subfamily: red, *Alphaherpesvirinae;* blue, *Betaherpesvirinae;* green, *Gammaherpesvirinae.* Statistically significant Nemenyi *post-hocs* after the Kruskal–Wallis test are reported. (B) Boxplot representation as in panel (A) for some representative species of the *Simplexvirus* genus. NHP simplexviruses are shown in orange, other mammal simplexviruses are shown in yellow (see Supplementary Table S2). (C) Scatter plot of the fraction of disordered residues for HSV-2 and VZV. Each dot represents an orthologous core protein.

Given the results above, we investigate whether a similar proteome fraction is occupied by IDRs in HSV-2 and in other HHVs. We thus used IUPred2 to calculate the fraction of disordered residues in the proteomes of HSV-2 and of other human alphaherpesviruses (HSV-1 and VZV), betaherpesviruses (HHV-6B and HCMV), and gammaherpesviruses (EBV and HHV-8). Results indicated that a significantly higher portion of simplexvirus proteins is disordered compared with the proteins of other HHVs (Fig. 4A). Among these latter, HHV-6B and HHV-8 showed the lowest fraction of IDRs (Fig. 4A).

We next assessed whether a high fraction of disordered residues is a general feature of simplexviruses by analyzing the proteomes of viruses that infect NHPs and other mammals. We found that the IDR fraction of NHP simplexvirus proteins is similar to that of HSV-1 and -2. Conversely, simplexviruses infecting other mammals tended to have a lower fraction of their proteomes occupied by disordered residues, although the difference with the human viruses was not statistically significant (Fig. 4B).

Because both HSV-1/-2 and VZV belong to the *Alphaherpesvirinae* subfamily but display very different fractions of IDRs, we compared HSV-2 and VZV orthologous core genes in terms of disordered fraction in the encoded proteins. With the exception of a few proteins with similar fraction of IDRs, most HSV-2 proteins showed a higher fraction of disordered residues than VZV proteins, indicating that the results we obtained at the level of the whole proteome (Fig. 4C) are not due to a minority of outliers. Very similar results were obtained when HSV-1 and VZV orthologs were analyzed (Supplementary Fig. S2).

### 3.3 Interaction with Host Proteins Influences the Disordered Fraction of Simplexvirus Proteins

**Figure 5.**
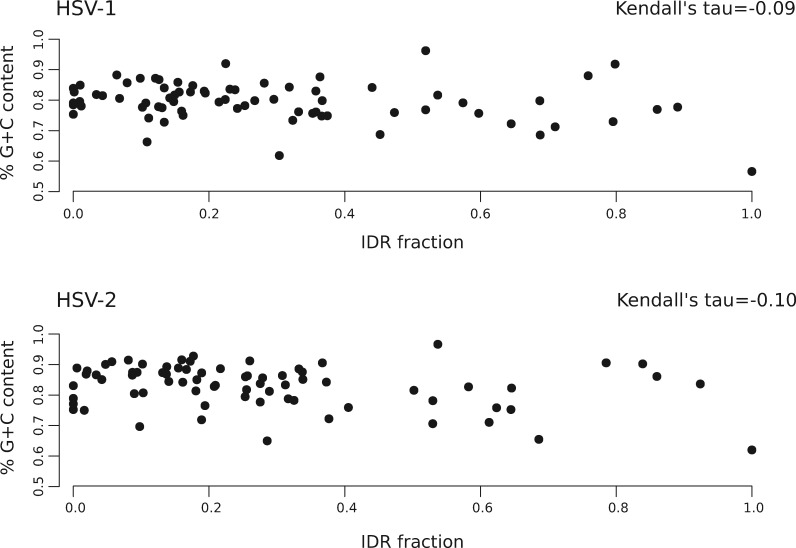
Correlation between G + C content and disordered residues. The percentage of G + C content calculated for the third codon position of each HSV-1 or -2 gene is plotted against the fraction of disordered residues for the protein encoded by the same gene.

Previous studies showed that the level of protein disorder in viral proteomes is influenced by genome size and base composition (G + C content; Pushker et al. 2013). We noticed no association between IDR fraction and genome size for HHV or HSVs that infect NHPs and other mammals (Fig. 4A and B). Primate simplexviruses, however, had higher G + C content (both calculated over all codon positions and for the third position only) compared with all other HHVs. Simplexviruses infecting non-primate mammals had intermediate G + C levels. Other than this effect, we observed no co-variation between G + C content and IDR fraction. For instance, the relatively G + C rich HCMV and EBV genomes encode proteins with a similar proportion of IDRs as the G + C poor HHV-6B and VZV genomes (Fig. 4A). To further address the role of base composition as a determinant of protein disorder in Simplexviruses, we correlated the IDR fraction of all HSV-1 and -2 proteins with the G + C content at the third codon position of their respective ORFs. No correlation was observed (Fig. 5), suggesting that the abundance of disordered regions in these viruses is not simply a consequence of their having G + C rich genomes.

**Figure 6.**
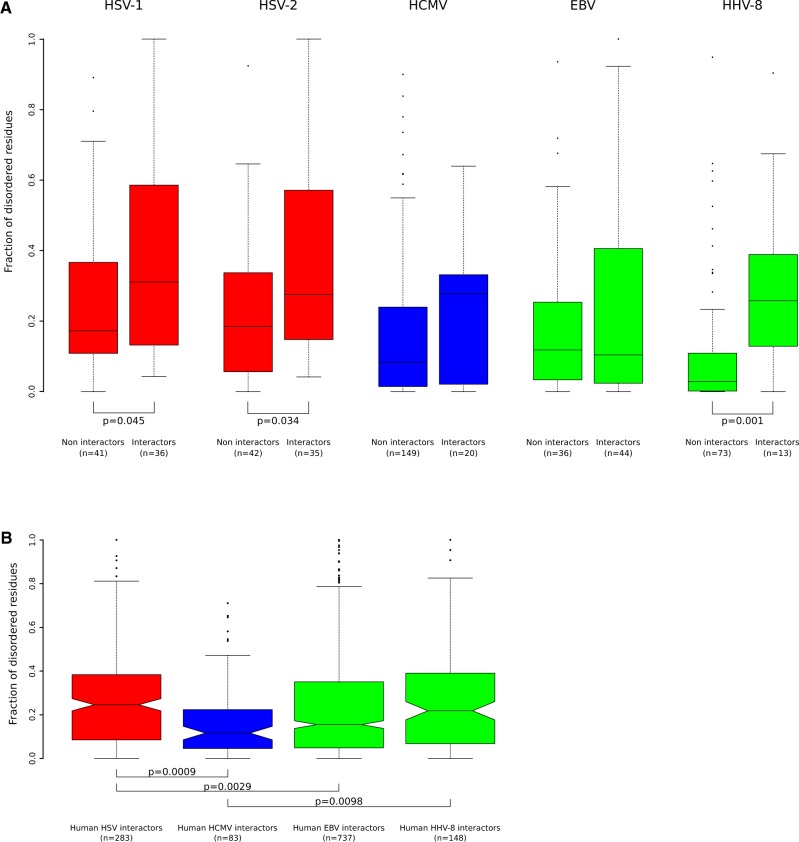
Fraction of disordered residues among interacting proteins. (A) Boxplots of the fraction of disordered residues for viral proteins having or not at least one known human protein interactor (see Section 2 for details). Statistically significant Wilcoxon rank sum tests are also reported. Colors are as in Fig. 4. (B) Fraction of disordered residues for human proteins that interact with herpesvirus proteins. Colors reflect panel (A). Statistically significant Nemenyi *post-hocs* after the Kruskal–Wallis test are reported.

Because IDRs are frequently involved in protein–protein interactions, we hypothesized that HHV proteins that interact with human factors have a higher fraction of disordered regions. Human–virus interactions were obtained from virus Mentha (https://virusmentha.uniroma2.it/;Calderone, Licata, and Cesareni 2015). As few interactions were available for HSV-2, and because HSV-1 and -2 are closely related, we assigned to both viruses the interactions reported for either. Conversely, HHV-6B and VZV were not analyzed, as very limited interaction data were available. Results indicated that the fraction of disordered residues is significantly higher in viral proteins that interact with human factors for HSV-1, -2, and HHV-8. This was not the case for EBV and HCMV (Fig. 6A).

### 3.4 Human interactors of simplexvirus proteins have a high fraction of disordered regions

Previous works have shown that human proteins that interact with viruses have a high fraction of IDRs, although differences were evident depending on the virus (Lou et al. 2016; Bosl et al. 2019). We wanted to investigate whether the different representation of IDRs in herpesvirus proteins is paralleled by different levels of disorder in the human interactors. We thus calculated IDRs for all herpesvirus-interacting human proteins, as derived from virus Mentha. Analysis indicated that indeed this is the case, as a significantly higher fraction of human proteins that interact with HSV-1 and -2 is occupied by IDRs compared with proteins that interact with EBV and HCMV products (Fig. 6B). Human interactors of HHV-8 proteins also displayed a consistent fraction of IDRs, although not as high as for HSV-1/-2 (Fig. 6B). These results were also confirmed when only interactors specific for each virus were analyzed (Supplementary Fig. S3).

## 4. Discussion

We exploited the peculiar evolutionary history of HSV-2 to investigate the relatively recent adaptive events that turned a chimpanzee herpesvirus into a successful human pathogen. To provide a genome-wide picture of the selective patterns acting on coding genes, we calculated the distribution of selection coefficients using gammaMap, which is relatively insensitive to the effects of demography and recombination (Wilson et al. 2011). As expected, we found that the majority of genes evolved under purifying selection. However, our major interest was to identify variants that increased in frequency as a result of positive selection after the cross-species transmission event. Such variants are expected to represent an adaptation to replication in human cells and to transmission in human populations. The most striking observation was the distribution of positively selected sites, as most of them occurred within IDRs. The clustering of selection signals, we observed in these regions, as well as in ordered portions, is expected to be minimally due to the approach we used, as the gammaMap sliding window model we applied (as recommended) only causes a slight increase in the probability of positive selection at nearby sites (Wilson et al. 2011). In any case, the significant enrichment of positive selection signals is independent of clustering, as we calculated the probability of IDRs and of non-disordered regions to harbor at least one positively selected site.

The general tendency of IDRs to be fast evolving was previously reported (Brown, Johnson, and Daughdrill 2010; Schlessinger et al. 2011; Toth-Petroczy and Tawfik 2011), and large-scale analyses in budding yeast and mammals indicated that significantly stronger positive selection is observed in intrinsically disordered compared with ordered regions (Nilsson, Grahn, and Wright 2011; Afanasyeva et al. 2018). Although to our knowledge, no systematic analysis was performed for viral proteomes, instances of adaptive evolution involving IDRs were described for some RNA viruses (Ortiz et al. 2013; Gitlin et al. 2014; Charon et al. 2018) and for polyomaviruses (Lauber et al. 2015). In particular, an analysis of the nodavirus polymerase indicated that the fast evolving, highly disordered C-terminus displays high functional robustness to amino acid replacements (Gitlin et al. 2014), whereas a study on the potyvirus genome-linked protein showed that amino acid changes that increase disorder expand the host range (Charon et al. 2018). Thus, disordered regions in viral proteins were suggested to afford evolutionary plasticity while preserving protein function (Gitlin et al. 2014; Charon et al. 2018). Our data strongly support this view and provide proteome-wide evidence that HSV-2 adaptation to the human/hominid host mainly occurred through changes within IDRs.

Previous studies indicated that, compared with cellular organisms, viral proteomes have a wider variation in IDR fraction (Pushker et al. 2013; Xue et al. 2014; Peng et al. 2015). Such variation only weakly depends on genome size, whereas a stronger effect was described for base composition. This is partially expected, as residues enriched in disordered regions are mainly encoded by G and C rich codons (Pushker et al. 2013; Basile et al. 2019). Clearly, this observation opens the question as to whether the fraction of IDRs is simply a consequence of base composition or if it is an adaptive feature driven by specific viral/host characteristics. Results herein document a high variability of disordered regions within the *Herpesviridae* family. We confirm that genome size is not a major determinant of the extent of protein disorder in herpesviruses and we show that base composition is also unlikely to explain the high fraction of simplexvirus proteomes occupied by IDRs. Although simplexviruses have G + C rich genomes, we found no correlation between protein disorder and codon composition at the third codon position. Instead, we show that non-core genes, most of which are involved in determining cellular tropism, immune evasion, and transactivation (Davison, Dargan, and Stow 2002), encode proteins with a higher fraction of IDRs than core genes. Consistently, viral proteins that interact with host components are more disordered than those without known human interactors. Notably, this effect was also observed for HHV-8 proteins: although the virus encodes proteins with a low IDR fraction, viral proteins that interact with the host have an extent of disorder comparable with that of HSV-1 and -2. These observations clearly point to the fact that viral protein function, rather than genome base composition, determines the fraction IDRs. This conclusion is strengthened by the observation that human proteins that interact with HSV-1/-2 also have a higher fraction of disorder than those interacting with EBV or HCMV, with HHV-8 interactors having intermediate levels of disorder. Thus, although human proteins that interact with viruses are, in general, more disordered than those with no viral binding partners (Lou et al. 2016; Bosl et al. 2019), differences exist depending on the virus and on the viral proteome. Indeed, a low level of disorder was previously reported for EBV interactors (Bosl et al. 2019). Conversely, the IDR fraction of human proteins that interact with other HHVs had never been investigated before. Interestingly, though, Lou and coworkers recently identified the DNA repair protein Nbs1 as an interactor of the HSV-1 protein ICP0 (encoded by *RL2*; Lou et al. 2016). Specifically, human Nbs1 binds the viral protein through a disordered region, and differences in this same region across primate species account for the species-specific effect of Nbs1 on viral replication. The authors thus suggested that, because of the fast evolution of IDRs, genetic arms-races between hosts and viruses may commonly involve disordered regions (Lou et al. 2016), as these are likely to form interaction surfaces and thus are expected to experience the strongest selective pressure (Sironi et al. 2015). Our data indirectly confirm this prediction for simplexviruses, as we found that HSV-2 adaptation to its human host mostly involved disordered regions, which are, in turn, more abundant among viral proteins that interact with host components. We also found that the average extent of disorder in viral proteins tends to parallel that of the host interactors, which is consistent with the genetic conflict hypothesis, as IDRs also evolve faster in humans and mammals (Afanasyeva et al. 2018). Thus, these data suggest that viruses that interact with fast-evolving, disordered human proteins in turn evolve disordered viral interactors poised for innovation.

Of course, a major open question concerns whether the marked differences in disordered fraction of herpesvirus proteomes reflect specific viral features. An interesting possibility is that the fraction of disorder in the viral proteome contributes to determine viral host range in terms of animal species and/or cell type. In fact, although most natural herpesvirus infections are species-specific, *in vitro* experiments and accidental cross-species transmission events indicate that alphaherpesviruses have a broader host range than betaherpesviruses and gammaherpesvirsus (Spear and Longnecker 2003; Azab et al. 2018). In the case of HCMV and EBV, post-cell entry events play a major role in limiting the infection to our species, indicating that these viruses cannot efficiently hijack the cellular machinery of non-human cells to promote their replication (Fioretti et al. 1973; Lafemina and Hayward 1988; Ellsmore, Reid, and Stow 2003; Jurak and Brune 2006; Schumacher et al. 2010; Muhe and Wang 2015). Among alphaherpesvirsus, VZV, with a low fraction of IDRs, is an exception, showing high human-specificity and the ability to infect few cell types (Zerboni et al. 2014). Conversely, HSV-1 and -2 can infect a variety of cell types from different mammals (Karasneh and Shukla 2011). Indeed, *in vivo* models of HSV-1/-2 pathogenesis were developed in rodents and NHPs (Kollias et al. 2015), and the accidental transmission to Old World as well as New World monkeys was documented (Azab et al. 2018). Thus, the high fraction of disordered protein regions in simplexvirus proteomes may provide flexibility in terms of cellular binding partners, possibly affording a wider host range.

## Data availability

All sequences used in this article are publicly accessible through the NCBI database. The GenBank Accession numbers of all sequences used in this article are available in Supplementary Material.

## Funding

This work was supported by the Italian Ministry of Health (‘Ricerca Corrente 2019–20’ to M.S. and ‘Ricerca Corrente 2018–20’ to D.F.*)*.

**Conflict of interest:** None declared. 

## Supplementary Material

veaa028_Supplementary_Data
