# Supplementary material for: Intrinsically disordered regions are abundant in simplexvirus proteomes and display signatures of positive selection
Source: Virus Evol. 2020 May 10;6(1):veaa028. doi: 10.1093/ve/veaa028 (PMC7211401; doi:10.1093/ve/veaa028)
Supplement: veaa028_Supplementary_Data [file veaa028_supplementary_data.pdf]

Supplementary Information

Intrinsically disordered regions are abundant in simplexvirus proteomes and display signatures of positive selection

Alessandra Mozzi[1*], Diego Forni[1], Rachele Cagliani[1], Mario Clerici[2,3], Uberto Pozzoli[1], Manuela Sironi[1]

[1] Scientific Institute, IRCCS E. MEDEA, Bioinformatics, 23842 Bosisio Parini, Italy.
[2] Department of Physiopathology and Transplantation, University of Milan, 20090 Milan, Italy.
[3] Don C. Gnocchi Foundation ONLUS, IRCCS, 20148 Milan, Italy.

* To whom correspondence should be addressed. Tel: +39-031877826; Fax: +39-031877499; Email:

alessandra.mozzi@lanostrafamiglia.it

Supplementary Tables:

Supplementary Table S1. List of HSV-2 strains used for gammaMap analysis.
Supplementary Table S2. List of herpesvirus strains used for intrinsic disorder analysis.
Supplementary Table S3. Positively selected sites detected by gammaMap analysis.

Supplementary Figures:

Supplementary Figure S1. Comparison between γ and dN-dS values.
Supplementary Figure S2. Fraction of disordered residues in VZV and HSV1.
Supplementary Figure S3. Fraction of disordered residues among human proteins.

Supplementary Table S1. List of HSV-2 strains used for gammaMap analysis.

Strain Name	GenBank ID	Length	Date	Country	Continent
2009-2222	MF510299	152219	-	Botswana	Africa
Isolate 17	LT797636	138913	2006/2013	Burundi	Africa
2006-17607	MF510281	152517	2006	Cameroon	Africa
Isolate 13	LT797623	138873	2006/2013	DRC	Africa
isolate 10	LT797629	138972	2006/2013	DRC	Africa
isolate 1	LT797627	138912	2006/2013	Guinea	Africa
Isolate 16	LT797624	138927	2006/2013	Guinea	Africa
Isolate 12	LT797633	138986	2006/2013	Ivory Coast	Africa
Isolate 9	LT799380	138917	2006/2013	Ivory Coast	Africa
2008-15116	MF621257	153414	-	Kenya	Africa
2009-4550	KX574893	153780	2008	Kenya	Africa
Isolate 7	LT797626	138969	2006/2013	Mali	Africa
Isolate 8	LT797625	138944	2006/2013	Mali	Africa
Isolate 3	LT797631	138962	2006/2013	Nigeria	Africa
Isolate 2	LT797622	138923	2006/2013	Niger	Africa
2009-3532	KX574892	153755	2007	South Africa	Africa
HSV-2/ZA/BID-G19093/SD66/1995	KR135320	153524	1995	South Africa	Africa
2009-3488	MF510347	151663	-	Tanzania	Africa
2009-409	KX574902	153721	2006	Tanzania	Africa
2011-34727	KX574899	153795	2011	Uganda	Africa
HSV-2/UG/BID-G19077/K39924/2007	KR135305	153108	2007	Uganda	Africa
2006-29060	KX574864	153024	2005	Zambia	Africa
2009-2265	MF510361	153281	-	Zambia	Africa
2006-21832	KX574863	153981	2005	Zimbabwe	Africa
2007-38120	KX574871	154126	2004	Zimbabwe	Africa
HSV-2/JP/BID-G32586/JA1	KR135322	153923	-	Japan	Asia
HSV-2/JP/BID-G32587/JA2	KR135323	153745	-	Japan	Asia
HSV-2/JP/BID-G32588/JA3	KR135324	154404	-	Japan	Asia
HSV-2/JP/BID-G32589/JA5	KR135325	154491	-	Japan	Asia
HSV-2/JP/BID-G32590/JA6	KR135326	153510	-	Japan	Asia
HSV-2/JP/BID-G32591/JA7	KR135327	153449	-	Japan	Asia
HSV-2/JP/BID-G32592/JA8	KR135328	153901	-	Japan	Asia
HSV-2/JP/BID-G32593/JA9	KR135329	153554	-	Japan	Asia
HSV2-H12211	KY922725	155111	-	Finland	Europe
HSV2-H12212	KY922726	155788	-	Finland	Europe
HSV2-H1226	KY922720	152981	-	Finland	Europe
HSV2-H1227	KY922721	152782	-	Finland	Europe
HSV2-H1229	KY922722	154136	-	Finland	Europe
HSV2-H1421	KY922723	148702	-	Finland	Europe
HSV2-H1526	KY922724	154678	-	Finland	Europe
Isolate 16	LT797786	138939	2006/2013	France	Europe
Isolate 14	LT797634	138854	2006/2013	France *	Europe
2006-49895	KX574868	153762	1994	USA	North America
2007-22031	KX574870	153679	2007	USA	North America
2010-6416	KX574896	153839	2010	USA	North America
2011-21761	KX574897	153501	1998	USA	North America

2012-32825	KX574901	153048	2012	USA	North America
Isolate 15	LT797630	138836	2006/2013	Martinique	South America
2004-4373	KX574861	153033	2003	Peru	South America
2006-30839	KX574865	153726	2005	Peru	South America
2007-38205	KX574860	153781	2004	Peru	South America
2007-38910	KX574877	153684	2006	Peru	South America
2007-39303	KX574878	153832	2007	Peru	South America

* African origin.

Supplementary Table S2. List of herpesvirus strains used for intrinsic disorder analysis.

Strain name	Accession ID	Virus species (Common Name, Abbreviation)	Subfamily	Genus	Host
Human herpesvirus 1 strain 17	NC_001806	Human alphaherpesvirus 1 (Herpes Simplex Virus 1, HSV-1)	*Alphaherpesvirinae*	Simplexvirus	Human
Human herpesvirus 2 strain HG52	NC_001798	Human alphaherpesvirus 2 (Herpes Simplex Virus 2, HSV-2)	*Alphaherpesvirinae*	Simplexvirus	Human
Chimpanzee alpha-1 herpesvirus strain 105640	NC_023677	Panine alphaherpesvirus 3 (Chimpanzee herpesvirus, PanHV-3)	*Alphaherpesvirinae*	Simplexvirus	Chimpanzee
Papiine herpesvirus 2 strain X313	NC_007653	Papiine alphaherpesvirus 2 (Herpesvirus papio 2, PaHV-2)	*Alphaherpesvirinae*	Simplexvirus	Yellow baboon
Macacine herpesvirus 1 strain E2490	NC_004812	Macacine alphaherpesvirus 1 (Macacine B virus, McHV-1)	*Alphaherpesvirinae*	Simplexvirus	Rhesus macaque
Cercopithecine alphaherpesvirus 2 SA8	NC_006560	Cercopithecine alphaherpesvirus 2 (Simian agent 8, CeHV-2)	*Alphaherpesvirinae*	Simplexvirus	Vervet monkey
Ateline alphaherpesvirus 1 isolate Lennette	NC_034446	Ateline alphaherpesvirus 1 (Herpesvirus ateles, AtHV-1)	*Alphaherpesvirinae*	Simplexvirus	Spider monkeys
Saimiriine herpesvirus 1 strain MV 5-4	NC_014567	Saimiriine alphaherpesvirus 1 (Saimiriine herpesvirus 1, SaHV-1)	*Alphaherpesvirinae*	Simplexvirus	Marmoset
Macropodid herpesvirus 1 isolate 3076/08	NC_029132	Macropodid herpesvirus 1 (Macropodid herpesvirus 1, MaHV-1)	*Alphaherpesvirinae*	Simplexvirus	Parma wallaby
Leporid herpesvirus 4 isolate LHV4012612	NC_029311	Leporid herpesvirus 4 (Leporid herpesvirus 4, LeHV-4)	*Alphaherpesvirinae*	Simplexvirus	Rabbit
Fruit bat alphaherpesvirus 1 DNA	NC_024306	Pteropodid alphaherpesvirus 1 (Fruit bat herpesvirus 1, PtAHV-1)	*Alphaherpesvirinae*	Simplexvirus	Fruit bat
Human herpesvirus 5 strain Merlin	NC_006273	Human betaherpesvirus 5 (Human Cytomegalovirus, HCMV)	*Betaherpesvirinae*	Cytomegalovirus	Human
Human herpesvirus 6B strain Z29	NC_000898	Human betaherpesvirus 6B (Human herpes virus 6B, HHV-6B)	*Betaherpesvirinae*	Roseolovirus	Human
Human herpesvirus 4 type 2 strain AG876	NC_009334	Human gammaherpesvirus 4 (Epstein-Barr virus type 2, EBV)	*Gammaherpesvirinae*	Lymphocryptovirus	Human
Human herpesvirus 8 strain GK18	NC_009333	Human gammaherpesvirus 8 (Kaposi's sarcoma-associated herpesvirus, HHV-8)	*Gammaherpesvirinae*	Rhadinovirus	Human

Supplementary Table S3. Positively selected sites detected by gammaMap analysis.

Gene	Position[a]	Ancestral codon	Derived codon	Ancestral AA	Derived AA	Minor allele frequency	Pr[b]
RL1	231	ATC	GCC	Ile	Ala	0	0.81
	233	GGA	GAA	Gly	Glu	0	0.8
	234	CCC	GAC	Pro	Asp	0	0.82
	242	GCG	GAG	Ala	Glu	0	0.76
	243	AGC	GCG	Ser	Ala	0.03	0.86
	244	GGG	GCG	Gly	Ala	0	0.84
	245	TCC	GCG	Ser	Ala	0	0.84
	247	GGG	GCG	Gly	Ala	0	0.81
RL2	78	ACC	GCG	Thr	Ala	0	0.8
	83	ATG	GCG	Met	Ala	0	0.90
	84	ACC	GCC	Thr	Ala	0	0.85
	169	AGA	CCG	Arg	Pro	0	0.97
	170	CTG	GTG	Leu	Val	0	0.9
	312	AGT	CCT	Ser	Pro	0	0.8
	399	AGT	GGA	Ser	Gly	0.03	0.97
	400	TTG	GGT	Leu	Gly	0	1
	403	TCA	CAG	Ser	Gln	0	1
	404	GAG	TCG	Glu	Ser	0.03	0.94
	499	GCC	GGT	Ala	Gly	0	0.89
	500	ACC	GCC	Thr	Ala	0.03	0.92
	501	ACC	GCC	Thr	Ala	0.08	0.94
	502	ACC	CCC	Thr	Pro	0.03	0.94
	503	GCC	CAC	Ala	His	0.06	0.94
RS1	186	GTG	ACG	Val	Thr	0	0.87
	187	CCG	TCC	Pro	Ser	0	0.88
	308	GCG	GAG	Ala	Glu	0	0.9
	309	ACC	CCC	Thr	Pro	0	0.96
	310	AAG	GCC	Lys	Ala	0	0.99
	311	GCC	CCC	Ala	Pro	0	0.99
	550	GGC	GAC	Gly	Asp	0	0.87
	553	GAG	GAC	Glu	Asp	0	0.94
	554	GAC	GCC	Asp	Ala	0	0.96
	555	GAC	AAC	Asp	Asn	0	0.97
	556	GGC	CGC	Gly	Arg	0	0.98
	557	GCC	CGC	Ala	Arg	0.37	0.99
	559	GCC	GGC	Ala	Gly	0	0.97
	561	GGG	GAC	Gly	Asp	0.09	1
	563	GCG	CGC	Ala	Arg	0	1
	565	CGC	AAG	Arg	Lys	0	1
	567	CGG	GCC	Arg	Ala	0	1
	679	GGG	GCG	Gly	Ala	0	0.93
	680	CCC	GGC	Pro	Gly	0	0.96
	681	ACG	GCG	Thr	Ala	0	0.96
	682	ACC	GCC	Thr	Ala	0	0.96

	819	GCG	CGC	Ala	Arg	0	1
	820	GGC	CCC	Gly	Pro	0	1
	822	CCG	GCC	Pro	Ala	0	1
	823	CGC	GCC	Arg	Ala	0	1
	825	GCG	CCC	Ala	Pro	0	1
	826	TCC	GCG	Ser	Ala	0	1
	827	CCG	GGG	Pro	Gly	0	1
	830	GGG	CCC	Gly	Pro	0	1
	831	GCC	CCC	Ala	Pro	0	1
	832	GAG	GCC	Glu	Ala	0	1
	835	GCC	ACG	Ala	Thr	0	0.91
UL12	5	GTG	GCA	Val	Ala	0	0.81
	6	GCA	ACA	Ala	Thr	0	0.82
	8	GAC	GGC	Asp	Gly	0	0.83
	9	ACC	GCC	Thr	Ala	0.07	0.83
	13	TCG	GCG	Ser	Ala	0	0.83
	17	TGC	CGC	Cys	Arg	0	0.86
	18	GTT	GAC	Val	Asp	0	0.87
	19	TCA	CCC	Ser	Pro	0	0.87
	20	GAA	GAT	Glu	Asp	0	0.86
	21	AAT	AGT	Asn	Ser	0.03	0.85
	24	CGG	AAG	Arg	Lys	0	0.79
	249	CAC	ACC	His	Thr	0	0.82
UL25	122	GGG	GAG	Gly	Glu	0	0.98
	123	CCC	GGC	Pro	Gly	0	1
	124	GAG	GCT	Glu	Ala	0	1
	125	GTG	GCG	Val	Ala	0	1
	126	GGC	GAC	Gly	Asp	0	1
	129	GAG	GCG	Glu	Ala	0	1
	130	GTT	CCG	Val	Pro	0	1
	134	TCG	GCG	Ser	Ala	0	0,91
	137	GTC	GCC	Val	Ala	0	0,83
UL26	548	GGG	CCT	Gly	Pro	0	1
	549	GCG	CTA	Ala	Leu	0	1
	550	GGG	TCC	Gly	Ser	0	1
	553	CCC	GTC	Pro	Val	0,03	0,94
	555	CGC	CCA	Arg	Pro	0	0,84
UL27	14	ACG	GCG	Thr	Ala	0	0,88
	16	ATG	GTG	Met	Val	0	0,89
	20	GTG	GCG	Val	Ala	0	0,89
	31	AGC	CGC	Ser	Arg	0	0,9
	32	CCC	GCC	Pro	Ala	0	0,9
	34	GCC	GGC	Ala	Gly	0	0,9
	38	GTG	GCG	Val	Ala	0	0,9
	40	CCT	GTC	Pro	Val	0	0,9
	44	GTG	GGG	Val	Gly	0,03	0,89
	45	AGT	GGT	Ser	Gly	0	0,89
	53	ATC	GTC	Ile	Val	0	0,86
	54	CAG	CCG	Gln	Pro	0	0,85

	64	CAG	CGG	Gln	Arg	0,25	0,8
	69	CAG	CCG	Gln	Pro	0	0,78
	75	CCG	GCG	Pro	Ala	0	0,76
UL33	83	AGG	GGG	Arg	Gly	0	0,78
	84	CAT	CCT	His	Pro	0	0,78
UL34	233	GCT	GGA	Ala	Gly	0	0,95
	234	GGT	CCG	Gly	Pro	0	0,98
	235	TCC	GCC	Ser	Ala	0	0,94
UL36	274	GCC	GTC	Ala	Val	0,02	0,78
	276	GCG	AGG	Ala	Arg	0	0,79
	278	GAG	GAC	Glu	Asp	0	0,77
	281	GCC	GGC	Ala	Gly	0	0,76
	291	ACA	GCG	Thr	Ala	0	0,75
	297	CGG	CAG	Arg	Gln	0	0,79
	299	CAA	CGG	Gln	Arg	0,13	0,88
	300	ACA	GCC	Thr	Ala	0	0,89
	303	GCC	CCC	Ala	Pro	0	0,93
	304	GCC	CCC	Ala	Pro	0	0,95
	305	TTG	GAG	Leu	Glu	0	0,95
	306	GTG	GCC	Val	Ala	0	0,95
	307	GCC	CCC	Ala	Pro	0	0,91
	310	GTC	GCC	Val	Ala	0	0,79
	311	CCG	TCG	Pro	Ser	0,03	0,77
	2618	GCC	GTC	Ala	Val	0	0,78
	2621	TCA	CAA	Ser	Gln	0	0,84
	2622	CAC	CAA	His	Gln	0,02	0,84
	2625	CGG	GGG	Arg	Gly	0	0,84
	2626	TCC	CGC	Ser	Arg	0	0,84
	2627	GCG	GTG	Ala	Val	0	0,83
	2628	CAC	CCC	His	Pro	0	0,8
UL43	285	CAC	TCT	His	Ser	0,05	1
	286	CGG	GAC	Arg	Asp	0	1
	287	GGG	GCG	Gly	Ala	0,07	1
	288	ACG	GCT	Thr	Ala	0	1
	289	ATG	CCG	Met	Pro	0	1
	290	CGA	TCA	Arg	Ser	0	1
UL44	64	CGA	CAA	Arg	Gln	0	0,84
	65	CAC	CCC	His	Pro	0	0,86
	66	CCC	CGC	Pro	Arg	0	0,87
	67	AAC	AAG	Asn	Lys	0	0,87
	68	ACG	GCG	Thr	Ala	0	0,86
	71	CAT	AGT	His	Ser	0	0,84
	194	CCA	GGT	Pro	Gly	0,31	1
	195	ACC	CGG	Thr	Arg	0,27	1
	335	GTG	ACG	Val	Thr	0	0,76
	386	GGA	ACA	Gly	Thr	0	0,8
UL46	570	TGC	GCC	Cys	Ala	0	0,83
	571	CTT	ATG	Leu	Met	0,03	0,82
	645	ACG	CCG	Thr	Pro	0	0,75

UL47	89	GGT	GCG	Gly	Ala	0	0.98
	90	CAC	GCG	His	Ala	0	0.99
	91	GGG	GCG	Gly	Ala	0	0.9
UL49	104	GGG	CCC	Gly	Pro	0	0.88
	109	GCC	GGC	Ala	Gly	0	0.87
	110	CCC	GCC	Pro	Ala	0	0.82
UL52	687	GTG	GCG	Val	Ala	0	0.82
	688	TCG	CCG	Ser	Pro	0	0.83
	690	GAA	GTC	Glu	Val	0	0.85
	691	CAC	TGT	His	Cys	0	0.84
US5	86	CCA	CAC	Pro	His	0	0.99
	87	CGC	GCG	Arg	Ala	0.14	1
	89	CCA	CAA	Pro	Gln	0	1
	90	ATT	TTT	Ile	Phe	0	1
	91	TGC	GCC	Cys	Ala	0	1
US6	368	CGA	CAG	Arg	Gln	0	0.82
	369	AAT	ATG	Asn	Met	0	0.85
	384	CAG	GCG	Gln	Ala	0	0.98
	385	ACA	CCC	Thr	Pro	0	0.98
US8A	77	CGG	CTG	Arg	Leu	0	0.79
	81	GAG	GCG	Glu	Ala	0	0.93
	82	GTG	GGC	Val	Gly	0	1
	83	CCG	GGC	Pro	Gly	0,02	1
	84	ACC	GCC	Thr	Ala	0	0,97
	85	GTG	GCG	Val	Ala	0,02	0,92
	89	CAC	CGC	His	Arg	0	0,78
	93	TAC	CGC	Tyr	Arg	0	0,98
	94	AGC	GGC	Ser	Gly	0	0,97
	95	TGC	GGC	Cys	Gly	0	0,95
US11	10	GCC	CCC	Ala	Pro	0	0.96
	11	CCA	CAA	Pro	Gln	0,06	0,97
	29	AAC	GAC	Asn	Asp	0	0,84

NOTES:
[a] Position refers to the HSV2 HG52 strain (NC_001798)
[b] Posterior probability of $\gamma \geq 1$, as detected by gammaMap

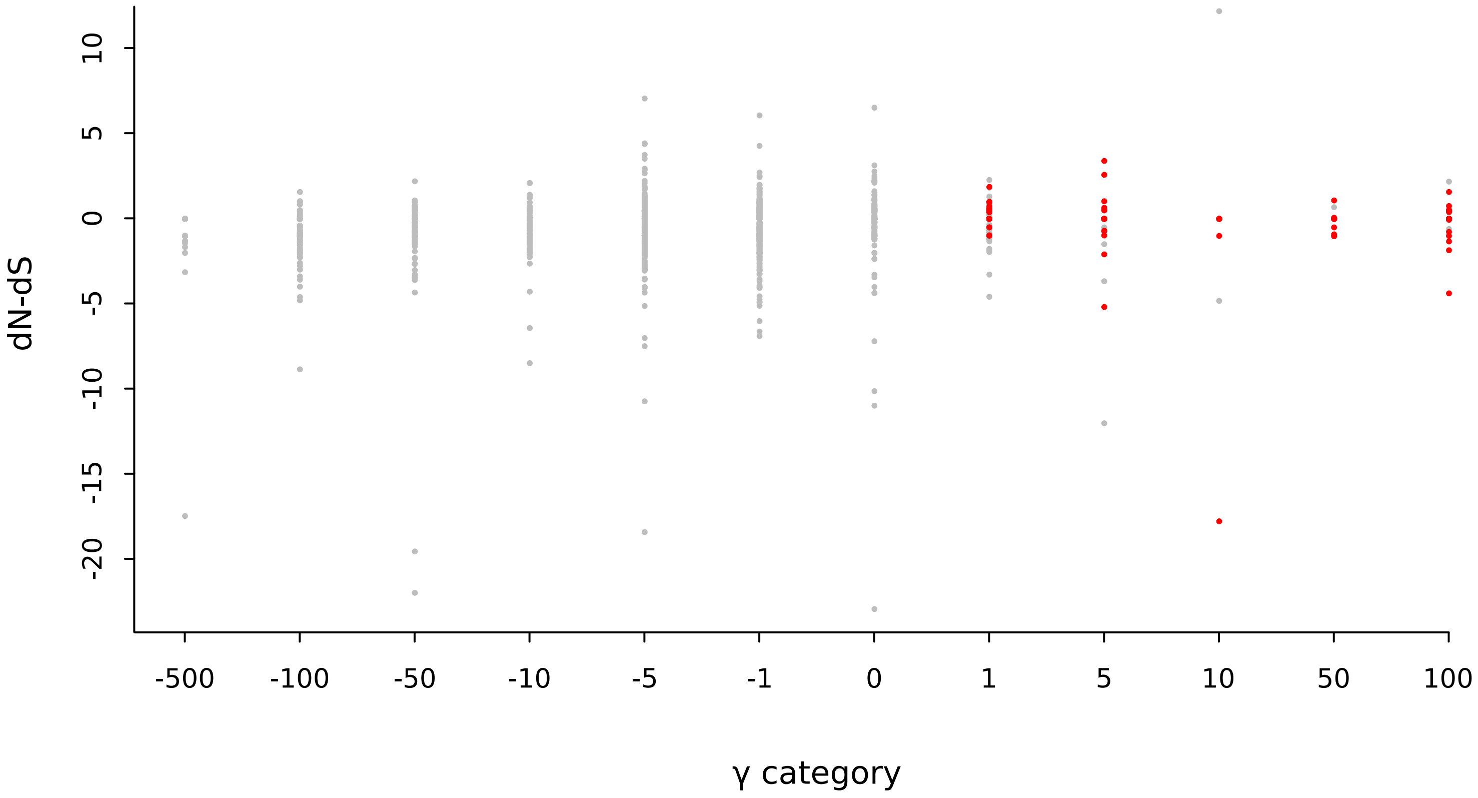

Supplementary Figure S1. Comparison between γ and dN-dS values. The most likely γ category is plotted against the dN-dS value for each codon of all genes having at least one positively selected site (red in the plot) defined by gammaMap (see Methods). Note that, although some sites had the highest posterior probability of γ >0, they were not called as positively selected because the cutoff we imposed (cumulative posterior probability of γ > 0 higher than 0.75) was not reached.

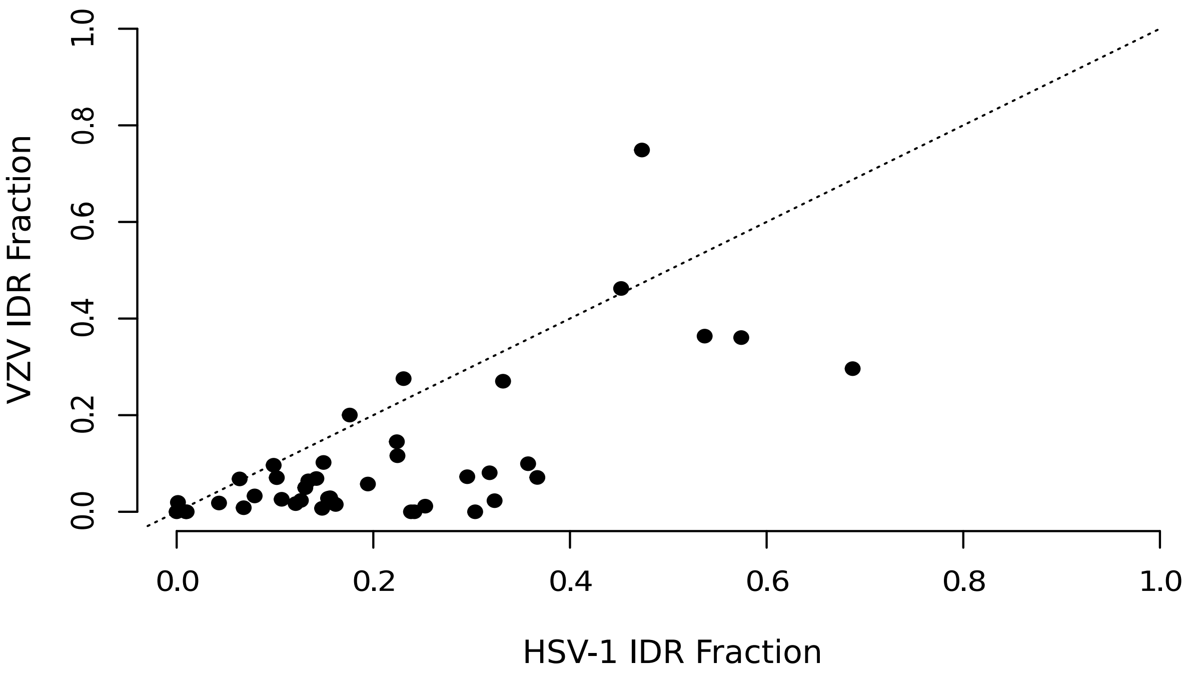

Supplementary Figure S2. Fraction of disordered residues in VZV and HSV-1. Scatter plot among fraction of disordered residues for HSV-1 and VZV. Each dot represents an orthologous *core* protein.

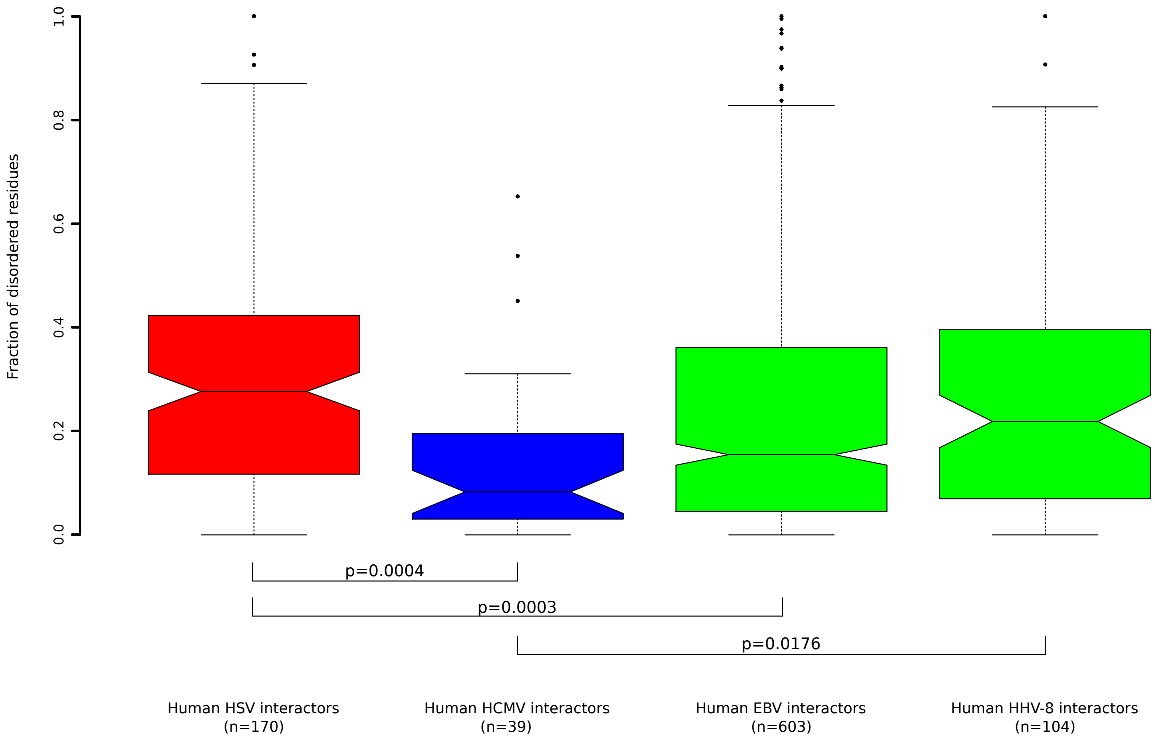

Supplementary Figure S3. Fraction of disordered residues among human proteins. IDR fraction for human proteins that interact with herpesvirus proteins. Human proteins that interact with proteins from different human herpesviruses were removed from the analysis. Statistically significant Nemenyi post-hoc after Kruskal-Wallis test are also reported.
